# Shikimic Acid Mitigates Deoxynivalenol-Induced Jejunal Barrier Injury in Mice via Activation of the Nrf-2/HO-1/NQO1 Pathway and Modulation of Gut Microbiota

**DOI:** 10.3390/antiox14101145

**Published:** 2025-09-23

**Authors:** Yijing Su, Bin Zheng, Chixiang Zhou, Miaochun Li, Yifeng Yuan, Han Wang, Bei Li, Shiyu Wu, Zhengkun Wu, Yinquan Zhao, Wei Zhang, Gang Shu

**Affiliations:** College of Veterinary Medicine, Sichuan Agricultural University, Chengdu 611130, China; yijingsu2003@163.com (Y.S.); zhengbin0229@163.com (B.Z.); 18080817059@163.com (C.Z.); limiaochun0813@163.com (M.L.); fcm0417@163.com (Z.W.); paprikazyq@163.com (Y.Z.); zhangwei26510c@126.com (W.Z.)

**Keywords:** deoxynivalenol, shikimic acid, intestinal barrier, oxidative stress, tight junction, gut microbiota

## Abstract

Deoxynivalenol (DON), a mycotoxin from Fusarium that contaminates cereals, can also induce intestinal injury. However, the mechanisms underlying DON-induced jejunal barrier injury remain unclear. This study demonstrates that shikimic acid (SA) alleviates DON-induced jejunal barrier damage and dysbiosis via antioxidant pathways. Fifty 5-week-aged male KM mice were divided into control (CON), model (MOD, 2.4 mg/kg bw DON), and SA-treated groups (LDG/MDG/HDG: 25/50/100 mg/kg bw SA + DON). After SA treatment, notably MDG, reversed DON-induced weight loss and jejunal hyperemia; ameliorated villus atrophy, crypt deepening and goblet cell loss, increasing villus/crypt ratio; reduced gut permeability markers (D-LA/DAO) and pro-inflammatory cytokines (TNF-α/IL-6/IL-1β); and dose-dependently upregulated tight junction proteins (ZO-1/Occludin/Claudin1). Mechanistically, SA activated the Nrf2/HO-1/NQO1 pathway, elevating antioxidants (GSH/SOD/AOC) while reducing MDA, with MDG showing optimal efficacy. 16S rRNA sequencing revealed MDG counteracted DON-induced dysbiosis by enriching beneficial bacteria (e.g., *Bacteroidota* at phylum level; *Muribaculaceae* at family level) and suppressing pathogens (*Staphylococcaceae*) (LDA score > 4.0). Thus, SA mitigates DON toxicity via Nrf2-mediated barrier restoration, anti-inflammation, and microbiota modulation. This research provides new insights for the further development of Shikimic Acid and the treatment of DON-induced jejunal barrier injury.

## 1. Introduction

Nuclear factor erythroid 2-related factor 2 (Nrf2) serves as the master regulator of antioxidant responses in mammals, governing the transcription of numerous antioxidant enzymes [1]. Under unstressed conditions, Nrf2 is sequestered by Kelch-like ECH-associated protein 1 (Keap1), directing it toward ubiquitination and proteasomal degradation. During oxidative stress, Nrf2 dissociates from Keap1, undergoes nuclear translocation, associates with antioxidant response elements (AREs), and initiates transcription of cytoprotective genes. Additionally, GSK-3β-mediated phosphorylation of Nrf2 promotes nuclear export, ubiquitination, and proteasomal degradation. Alternatively, active GSK-3β enables Fyn-mediated phosphorylation of Nrf2, triggering its nuclear export and degradation to terminate Nrf2-dependent antioxidant signaling [2].

Deoxynivalenol (DON), a type B trichothecene mycotoxin, is produced by Fusarium fungi. Classified among the five major agricultural mycotoxins [3], DON exposure induces reduced growth performance and gut microbiota dysbiosis in livestock like pigs and chickens [4,5], resulting in substantial economic losses within the livestock industry. Moreover, DON elicits adverse effects including diarrhea, vomiting, and gastrointestinal inflammation, posing risks to both human and animal health [6]. Recent surveillance data indicate that DON contaminates over 60% of tested food samples at levels above safety limits [7], and exposure-related toxicoses are emerging as an increasing public health concern [6]. Consequently, the development of effective strategies to mitigate the detrimental effects of DON on intestinal health is imperative for the livestock sector. Previous research on DON has documented its severe toxicological profile, encompassing carcinogenic, teratogenic, and inflammatory effects [8]. with its primary mechanisms involving disruption of the intestinal barrier, impairment of tight junctions, and induction of oxidative stress in intestinal tissues [9,10]. Studies have also demonstrated that DON-induced oxidative stress can be ameliorated by activating the Nrf2/HO-1 pathway and its related signaling pathways [11,12]. The jejunum represents the primary site of intestinal DON absorption [13], a key region for dietary lipid processing and host-microbiome interactions [14], playing a pivotal role in animal growth, development, and health. However, research specifically examining the effects of DON exposure on jejunal tissue remains scarce. Therefore, focusing on jejunal tissue offers a more comprehensive approach to elucidating DON toxicity.

Given the importance of oxidative stress in DON toxicity, natural compounds with antioxidant and anti-inflammatory properties may represent promising countermeasures. Shikimic acid (SA) is a naturally occurring chiral compound. SA functions as a key metabolic precursor to cinnamic acid, anthocyanins, flavonoids, tannins, essential aromatic amino acids (L-phenylalanine, L-tyrosine, L-tryptophan), lignans, and various plants and microbial alkaloids (e.g., quinine) [15]. SA exhibits potent antioxidant, anti-inflammatory, and anti-thrombotic activities, and in vitro studies demonstrate that SA inhibits the proliferation and migration of vascular smooth muscle cells (VSMCs), suggesting potential efficacy against occlusive vascular diseases [16,17,18,19]. And has been shown to improve tight junction integrity, suppress pro-inflammatory cytokine production, and modulate gut microbiota in models of intestinal inflammation, such as dextran sulfate sodium (DSS)-induced colitis, SA can suppress phosphorylation of critical proteins within the MAPK and NF-κB signaling pathways, leading to inhibition of inflammatory factor expression, enhancement of intestinal barrier function, amelioration of intestinal inflammation, and fortification of intestinal immunity. Collectively, these findings demonstrate the anti-inflammatory capabilities of SA [20]. Nevertheless, whether SA can protect against DON-induced jejunal injury and the underlying mechanisms remain unknown.

We hypothesize that SA can alleviate DON-induced jejunal barrier injury by activating the Nrf2/HO-1/NQO1 antioxidant signaling pathway and modulating gut microbiota composition.

## 2. Materials and Methods

### 2.1. Experimental Animal

A total of 50 male Kunming mice aged 5 weeks were used in the study. The mice were purchased from Pegatron Biotech (Chengdu, China). The use of animals was approved by the Sichuan Agricultural University Animal Ethical and Welfare Committee (approval No.: dyy-20240671). The mice were housed (5/cage) in an SPF facility, 12 h/12 h light–dark cycle, 22 ± 1 °C, 50–60% relative humidity, standard pellet diet and water ad libitum. Before experiments, the mice were fasted for 12 h with free access to food.

### 2.2. Chemicals

DON was obtained from Psaitong Biotechnology Co., Ltd. (Beijing, China). The purity of DON is 98% or higher, as stated by the manufacturer (Psaitong, Beijing, China). SA were obtained from (Layn Natural Ingredients Corp, Guilin, China). The purity of SA is 98% or higher, as stated by the manufacturer (Layn, Guilin).

### 2.3. Experimental Procedure

Following a 7-day acclimatization period, fifty mice were equally divided into five groups: control (CON), model (MOD, 2.4 mg/kg bw DON) [21], low-dose (LDG, 25 mg/kg bw SA + 2.4 mg/kg bw DON), medium-dose (MDG, 50 mg/kg bw SA + 2.4 mg/kg bw DON), and high-dose (HDG, 100 mg/kg bw SA [22] + 2.4 mg/kg bw DON). DON and SA were dissolved in purified water, with doses selected based on prior studies. During the initial 7-day phase, CON received distilled water by gavage, MOD received normal saline, and LDG/MDG/HDG received SA solutions at respective doses; in the final 7-day phase, CON continued with distilled water, MOD received DON solution, and LDG/MDG/HDG received mixed SA-DON solutions. All gavage administrations used a volume of 0.1 mL/10 g bw. Mice were euthanized at the end of the experiment. Blood samples were collected and centrifuged to obtain the serum. Jejunum tissues were fixed in 4% paraformaldehyde or 2.5% glutaraldehyde and also preserved at −80 °C.

### 2.4. HE Staining

Histomorphological analysis was performed by BIOSSCI Biotechnology Co., Ltd. (Wuhan, China), a qualified diagnostics provider, included preparation of paraffin-embedded sections and hematoxylin-eosin (HE) staining. Tissue sections were examined and imaged using optical microscopy. Villus length, crypt depth, and their ratio were quantified with ImageJ 2.16.0 software.

### 2.5. Biochemical Analysis

#### 2.5.1. Determination of T-AOC, MDA, GSH Levels and SOD Activities in Serum

Blood samples were centrifuged (5000 r, 15 min, 4 °C), and the supernatants were collected. Serum concentrations of total antioxidant capacity (T-AOC), superoxide dismutase (SOD), reduced glutathione (GSH), and malondialdehyde (MDA) were determined using commercial kits (A015-1-2, A001-3-2, A005-1-2, A003-1-2, Nanjing Jiancheng Bioengineering Institute, Nanjing, China) per manufacturer’s protocols. Absorbance readings were taken at specific wavelengths: 520 nm for T-AOC, 532 nm for MDA, 550 nm for SOD, and 405 nm for GSH using a Biotek microplate reader (Winooski, VT, USA). Data analysis incorporated results from six independent experimental replicates.

#### 2.5.2. Determination of DAO, D-LA Levels in Jejunum Tissue

Jejunal tissues were homogenized in physiological saline and centrifuged (3000 r, 10 min, 4 °C). Supernatants were collected for quantification of diamine oxidase (DAO) and D-lactate (D-LA) concentrations using ELISA kits (ml076356, ml895244, Shanghai Enzyme-linked Biotechnology Co., Ltd., Shanghai, China), following the manufacturer’s protocols. Absorbance measurements at 450 nm were conducted with a microplate reader (Diatek, Wuxi, China). Data analysis included six independent experimental replicates.

#### 2.5.3. Determination of TNF-a, IL-6, IL-1β Levels in Jejunum Tissue

Murine jejunal tissues were homogenized to prepare tissue homogenates. The levels of tumor necrosis factor-α (TNF-α), interleukin-1β (IL-1β), and interleukin-6 (IL-6) in the jejunal homogenates were quantified using specific ELISA kits (HY-H0007, HY-H0001, HY-H0019, Beijing Sino-UK Institute of Biological Technology, Beijing, China) according to the manufacturer’s instructions. Absorbance was measured at 450 nm using a microplate reader (Diarek, Wuxi, China). Statistical analyses were performed based on data from eight experiments.

### 2.6. Transmission Electron Microscope

In each group, 0.2 g of the jejunum was taken, immersed in glutaraldehyde, and preserved at 4 °C. Ultrathin sections were made from the preserved samples according to standard protocols. The ultrastructural changes were observed using a transmission electron microscope (Jeol, Tokyo, Japan, JEM-1400-FLASH).

### 2.7. Jejunum Tissue qRT-PCR Analysis

#### 2.7.1. RNA Acquisition and Primer Design

Intestinal RNA was extracted using Trizol reagent (Biomed, RA101-02). 0.1 g of intestinal tissue was placed in a grinding tube and mixed with 1 mL of Trizol. The mixture was then ground twice at 60 Hz for 60 s each, with a 10 s interval between grindings, so as to obtain a tissue homogenate sample. Centrifuge the sample (12,000 r, 5 min, 4 °C) and take 600 μL of supernatant. The supernatant was extracted with chloroform, isopropanol and 75% ethanol, and 20 μL ddH2O was added to obtain RNA. Primer design was performed at the National Center for Biotechnology Information, and BLAST (version 2.13.0+) and global alignment algorithms were used to avoid primer pairs that might lead to non-specific amplification. Specific primer information can be found in Table 1.

#### 2.7.2. Real-Time Quantitative PCR

The extracted RNA was subjected to reverse transcription reaction and transcribed into cDNA using reverse transcription kit (Biosharp, Tallin, Estonia, BL761A). Subsequently, a real-time fluorescent quantitative PCR reaction was performed by a two-step method using SYBR Green PCR Master Mix (Qiagen, Venlo, The Netherlands, Mat. No. 1129280). The specific conditions are: 50 °C for 2 min, 95 °C for 5 min, 95 °C for 5 s, 60 °C for 30 s, 95 °C for 15 s, 60 °C for 1 min, 40 cycles in the third and fourth steps, and shooting in the fourth step. Next, the melting curve was measured at an initial temperature of 60 °C, an end temperature of 90 °C, and an equilibrium time of 1 s. Eight replicates were set for each group to ensure data reliability. By comparing the Ct values, the relative expression of the target gene was calculated using the 2^−ΔΔCt^ method, and the normalized results were plotted.

### 2.8. Western Blot

Intestinal tissue samples were homogenized in an ice-cold mixture of RIPA buffer (Servicebio, G2002, Wuhan, China) and protease/phosphatase inhibitors (Biosharp, BL439A) for protein extraction. Protein concentration was determined using a BCA protein quantification kit (Biosharp, BL521A). Equal amounts of protein samples were separated by SDS-PAGE and subsequently transferred onto PVDF membranes. Following blocking with a protein-free rapid blocking solution (Servicebio, G2052) for 15 min, the membranes were incubated overnight at 4 °C with primary antibodies against the following targets: Nrf-2 (Proteintech, Rosemont, IL, USA, 16396-1-AP), Keap1 (Affinity, AF5266, Changzhou, China), NQO1 (Affinity, DF6437), HO-1 (Proteintech, 10701-1-AP), ZO-1 (Abmart, TA5145F, Shanghai, China), Occludin (Proteintech, 27260-1-AP), Claudin-1 (Affinity, AF0127), and β-actin (Cellcook, AR1146, Guangzhou, China). The next day, membranes were washed three times with TBST and incubated with an HRP-conjugated Goat Anti-Rabbit IgG (H+L) secondary antibody (Proteintech, SA00001-2) at room temperature for 1 h. After additional washes, protein bands were visualized using an ultra-sensitive ECL chemiluminescent substrate (Biosharp, BL523A). Band intensities were quantified via grayscale analysis using ImageJ software, normalized to β-actin expression, and subjected to statistical analysis based on four independent biological replicates.

### 2.9. Microbial 16S rRNA Gene Sequencing and Analysis

Cecal content samples were collected from mice under sterile conditions. Total genomic DNA was extracted using the Magnetic Soil and Stool DNA Kit (TIANGEN, Beijing, China, Catalog No. DP712) following the manufacturer’s instructions. The extracted DNA was quantified using a Qubit Fluorometer (Invitrogen, Austin, TX, USA) and assessed for purity by NanoDrop spectrophotometry (Thermo Scientific, Waltham, MA, USA). The V3–V4 hypervariable regions of the bacterial 16S rRNA gene were amplified with specific primers (341F:5′-CCTACGGGNGGCWGCAG-3′;806R:5′-GGACTACHVGGGTWTCTAAT-3′) with sample-specific barcodes. PCR amplification was performed using Phusion^®^ High-Fidelity PCR Master Mix (New England Biolabs, Ipswich, MA, USA). The PCR products were purified, quantified, and pooled in equimolar ratios. Sequencing libraries were constructed using the NEB Next^®^ Ultra™ II FS DNA PCR-free Library Prep Kit (New England Biolabs, USA) and paired-end sequencing (2 × 250 bp) was performed on the Illumina NovaSeq 6000 platform (Novogene, Tianjin, China).

Bioinformatic analysis was performed using QIIME2 (version 2022.2). Raw sequences were demultiplexed, quality-filtered, denoised, and merged using the DADA2 algorithm to obtain amplicon sequence variants (ASVs). Chimeric sequences were identified and removed using the UCHIME algorithm. Taxonomy was assigned to ASVs using the SILVA database (v138). To further analyze community structure, beta diversity distance matrices (weighted/unweighted UniFrac, Bray–Curtis) were calculated and visualized via principal coordinate analysis (PCoA) and non-metric multidimensional scaling (NMDS). Meanwhile, linear discriminant analysis effect size (LEfSe) was employed to identify differentially abundant taxa between groups.

### 2.10. Statistical Analysis

All data are presented as mean ± standard deviation (SD). Normality (Shapiro–Wilk test) and homogeneity of variance (Levene’s test) were confirmed for all datasets prior to parametric testing. Statistical analyses were performed using GraphPad Prism software (version 10.0). Changes in mouse weight were analyzed using two-way repeated-measures analysis of variance (ANOVA), with DON and SA administration as two between-subjects factors and time as the within-subjects repeated measure. Where significant effects were found, simple effects analysis followed by Bonferroni post hoc testing was applied for pairwise comparisons. For biochemical indicators, qPCR, and Western blot data, one-way ANOVA was used to assess group differences, followed by Tukey’s post hoc test for multiple comparisons. All graphs were generated using Prism 10.0. The significance threshold was set at *p* < 0.05, with *p* < 0.01 considered highly significant. In figures: *, **, ***, denote *p* < 0.05, <0.01, <0.001 versus the MOD; ^#^, ^##^, ^###^, denote *p* < 0.05, <0.01, <0.001 versus the CON.

## 3. Results

### 3.1. SA Mitigates DON-Induced Impairment of Mice Growth

**Figure 1:**
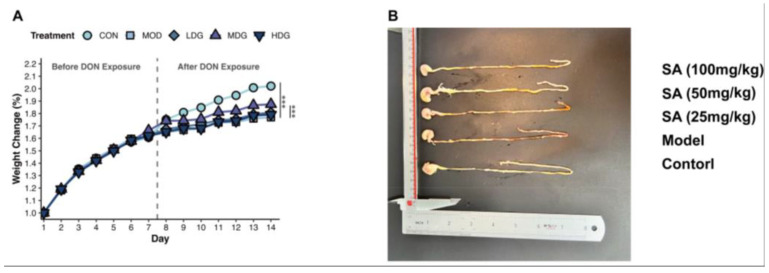
SA Mitigates DON-Induced Impairment of Mice Growth. (**A**) Mice growth curve and mice weight changes (*n* = 10). A two-way repeated-measures ANOVA was used for statistical analysis. (**B**) Displays a representative image of the jejunum. *** *p* < 0.001 vs. MOD.

To investigate whether SA mitigates the adverse effects of DON on mice growth rate, changes in body weight were monitored. Compared to the CON, the MOD, LDG, MDG, and HDG all exhibited varying degrees of weight reduction. From day one onwards, the weight change in the MOD was significantly lower than in the CON (*p* < 0.001), indicating that DON regulates mouse body weight. Notably, the weight change in the MDG group was significantly higher than in the MOD (*p* < 0.001; Figure 1A). After 14 days of feeding, mice were anesthetized and dissected. Compared to CON mice, the jejunum of DON-treated mice appeared markedly hyperemic. This hyperemia progressively diminished in the LDG, MDG, and HDG (Figure 1B).

### 3.2. SA Alleviates DON-Induced Damage to the Jejunal Intestinal Barrier in Mice

**Figure 2:**
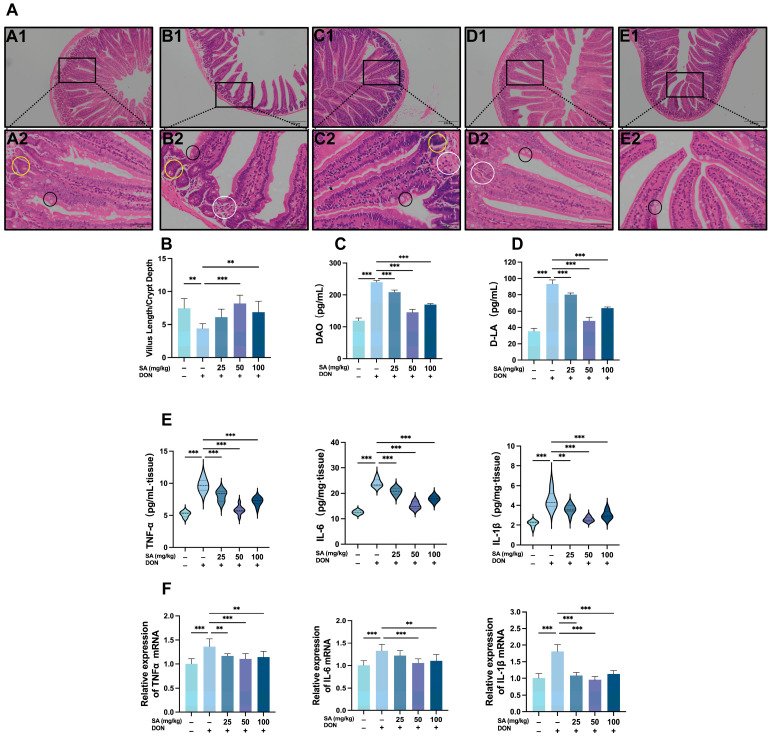
SA Alleviates DON-Induced Damage to the Jejunal Intestinal Barrier in Mice (One-Way ANOVA was used for all statistical analyses). (**A**) HE staining results for jejunum tissues of mice. (**A1**–**E1**) jejunum structure in 100-fold visual field, (**A2**–**E2**) jejunum structure in 400-fold visual field. (**A1**,**A2**) CON, (**B1**,**B2**) MOD, (**C1**,**C2**) LDG, (**D1**,**D2**) MDG, (**E1**,**E2**) HDG. Black circle: goblet cell. White circle: inflammatory cell infiltration. Yellow circle: crypt. (**B**) Results of DON and SA on ileal jejunum of mice. (**C**) Results of DON and SA on the content of DAO (*n* = 6). (**D**) Results of DON and SA on the content of D-LA (*n* = 6). (**E**) Results of DON and SA on the content of TNF-α, IL-6, IL-1β (*n* = 6). (**F**) Results of DON and SA on the mRNA content of TNF-α, IL-6, IL-1β (*n* = 8). **, *** *p* < 0.01, *p* < 0.001 vs. MOD.

HE staining of jejunal tissue revealed that compared to CON mice, DON-treated mice displayed distinct pathological alterations in the jejunum, including villus blunting and rupture, deepened crypts (Figure 2A(B1)), reduced goblet cell numbers, and inflammatory cell infiltration (Figure 2A(B2), white circle). Administration of SA significantly ameliorated these intestinal barrier defects and inflammatory manifestations (Figure 2A(A1)). The ratio of villus height to crypt depth (V/C ratio) serves as an indicator of growth rate. Measurement of villus height and crypt depth demonstrated that the V/C ratio was significantly reduced in the MOD compared to the CON (*p* < 0.01; Figure 2B). SA treatment significantly increased this ratio in the MDG (*p* < 0.001) and HDG (*p* < 0.01) groups compared to the MOD. These results indicate that DON damages jejunal villi and crypts, and SA attenuates this jejunal injury.

Compromised intestinal barrier integrity leads to increased release of D-LA and DAO into the blood and tissues; their levels reflect the extent of mucosal structural damage and increased intestinal barrier permeability. Quantitative analysis of D-LA and DAO in jejunal tissue showed that compared to the CON, levels of both D-LA (*p* < 0.001; Figure 2C) and DAO (*p* < 0.001; Figure 2D) were significantly elevated in the MOD. SA treatment at different doses significantly reduced D-LA and DAO levels compared to the MOD, with the most pronounced reduction observed in the MDG (*p* < 0.001).

Given the critical roles of TNF-α, IL-6, and IL-1β in mediating intestinal inflammation and barrier disruption, their levels in jejunal tissue were quantified as key indicators of barrier damage. Compared to the CON, the MOD exhibited significantly increased levels of TNF-α, IL-6, and IL-1β (*p* < 0.001; Figure 2E). Treatment with varying doses of SA significantly reduced the levels of these pro-inflammatory cytokines compared to the MOD, with the MDG showing the most substantial decrease (*p* < 0.001). Quantitative polymerase chain reaction (qPCR) analysis of these cytokines in jejunal tissue yielded consistent results with the ELISA findings (Figure 2F).

### 3.3. Effects of DON and SA on Tight Junctions in Mouse Jejunal Tissue

**Figure 3:**
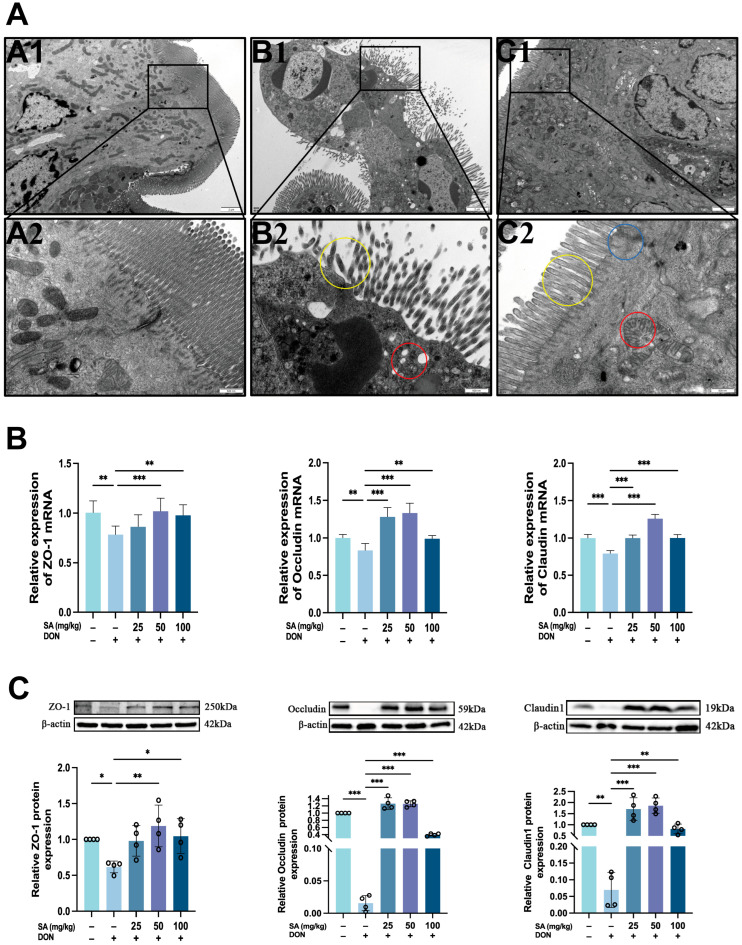
Effects of DON and SA on Tight Junctions in Mouse Jejunal Tissue (One-Way ANOVA was used for all statistical analyses). (**A**) Morphological changes in the microvilli and tight junction proteins in intestinal epithelial cells were observed by TEM. (**A1**–**C1**) ×4500, (**A2**–**C2**) ×25,000. (**A1**,**A2**) CON, (**B1**,**B2**) MOD, (**C1**,**C2**) MDG. Red circle: Swelling of Mitochondria; yellow circle: Sparse intestinal epithelial microvilli; blue circle: tight junction. (**B**) Result of DON and SA on the mRNA content of ZO-1, Occludin, and Claudin1. (**C**) Result of DON and SA on the relative content of tight junction proteins in mice (*n* = 4). *, **, *** *p* < 0.05, *p* < 0.01, *p* < 0.001 vs. MOD.

To systematically evaluate the effects of DON and SA on the ultrastructure of mouse jejunum, we performed transmission electron microscopy (TEM) analysis. DON treatment induced mitochondrial swelling (Figure 3A(B1), red circle), sparse and disorganized microvilli (Figure 3A(B2), yellow circle), and opened tight junctions. SA treatment ameliorated these changes, reducing mitochondrial swelling, restoring microvilli alignment, and normalizing tight junctions.

Tight junctions are crucial for maintaining the intestinal barrier, with ZO-1, Occludin, and Claudin-1 being key tight junction proteins. qPCR analysis of these proteins in jejunal tissue (Figure 3B) revealed that DON treatment significantly decreased the mRNA expression of ZO-1, Occludin, and Claudin-1 (*p* < 0.01, *p* < 0.01, *p* < 0.001, respectively). SA treatment rescued the expression of these genes in jejunal tissue, with the MDG showing the most significant effect (*p* < 0.001, *p* < 0.001, *p* < 0.001, respectively). Notably, ZO-1 mRNA expression in the LDG did not differ significantly from the MOD. Western blot analysis (Figure 3C) confirmed that DON treatment significantly reduced ZO-1, Occludin, and Claudin-1 protein levels (*p* < 0.05, *p* < 0.001, *p* < 0.01, respectively). SA treatment increased the expression of these proteins, with MDG again being most effective (*p* < 0.01, *p* < 0.001, *p* < 0.001, respectively). ZO-1 protein expression in the LDG did not differ significantly from the MOD.

### 3.4. SA Mitigates DON-Induced Jejunal Oxidative Stress in Mice by Enhancing the Nrf-2/HO-1/NQO1 Signaling Pathway

**Figure 4:**
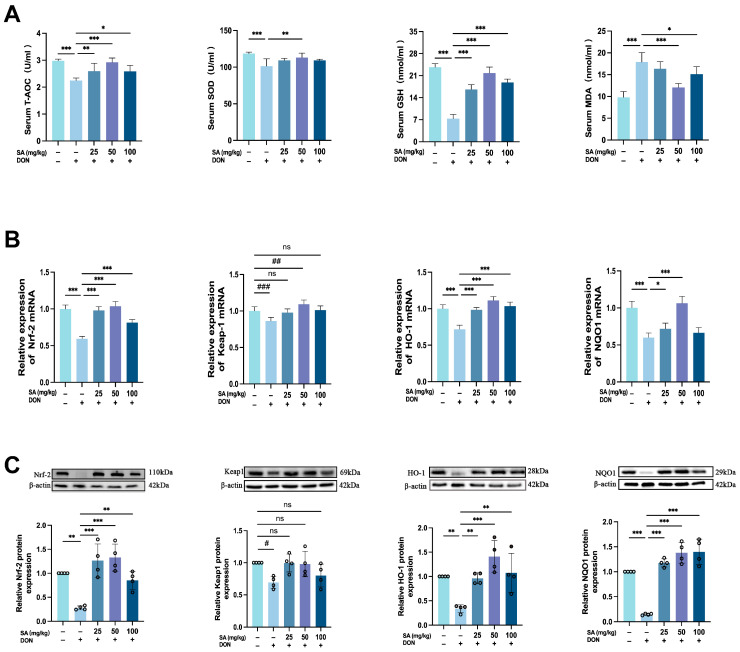
SA Mitigates DON-Induced Jejunal Oxidative Stress in Mice by Enhancing the Nrf-2/HO-1/NQO1 Signaling Pathway (One-Way ANOVA was used for all statistical analyses). (**A**) Result of DON and SA on the content of T-AOC, SOD, GSH, MDA. (**B)** Result of DON and SA on the mRNA content of Nrf-2, Keap1, HO-1, NQO1 (*n* = 8). (**C**) Result of DON and SA on the relative content of Nrf-2, Keap1, HO-1, NQO1 in mice (*n* = 4). *, **, *** *p* < 0.05, *p* < 0.01, *p* < 0.001 vs. MOD. ns, #, ##, ### *p* > 0.05, *p* < 0.05, *p* < 0.01, *p* < 0.001 vs. CON.

Given that DON induces significant oxidative stress in the mouse intestine, contributing to barrier damage, and considering SA’s known antioxidant properties, we evaluated whether SA administration could counteract DON-induced oxidative stress. We assessed the levels of GSH, MDA, SOD, and T-AOC in jejunal tissue (Figure 4A). DON treatment significantly decreased T-AOC, SOD, and GSH levels while increasing MDA levels (*p* < 0.001 for all). As anticipated, SA treatment increased T-AOC, SOD, and GSH levels and decreased MDA levels. The MDG exhibited the strongest effect (for T-AOC, SOD, GSH, and MDA, respectively: *p* < 0.0001, *p* < 0.01, *p* < 0.001, *p* < 0.001, respectively). However, SOD levels in the LDG and HDG did not differ significantly from the MOD.

The Nrf2/Keap1 signaling pathway plays a critical role in mediating the oxidative stress response. We evaluated the mRNA expression of key pathway components—nuclear factor erythroid 2-related factor 2 (Nrf2), Kelch-like ECH-associated protein 1 (Keap1), heme oxygenase-1 (HO-1), and NAD(P)H quinone oxidoreductase 1 (NQO1)—in jejunal tissues across groups. Results (Figure 4B) showed that compared to the CON, DON treatment significantly decreased the expression of Nrf2, Keap1, HO-1, and NQO1 (*p* < 0.001, *p* < 0.001, *p* < 0.001, *p* < 0.001, respectively). SA treatment significantly increased the expression of Nrf2, HO-1, and NQO1. However, Keap1 mRNA expression did not differ significantly from the CON, except in MDG (*p* < 0.01). Western blot analysis (Figure 4C) revealed similar trends for Nrf2, HO-1, and NQO1 protein expression. Keap1 protein expression did not differ significantly from CON, except in the MOD (*p* < 0.05).

### 3.5. Effect of SA on DON-Induced Gut Microbiota Dysbiosis

**Figure 5:**
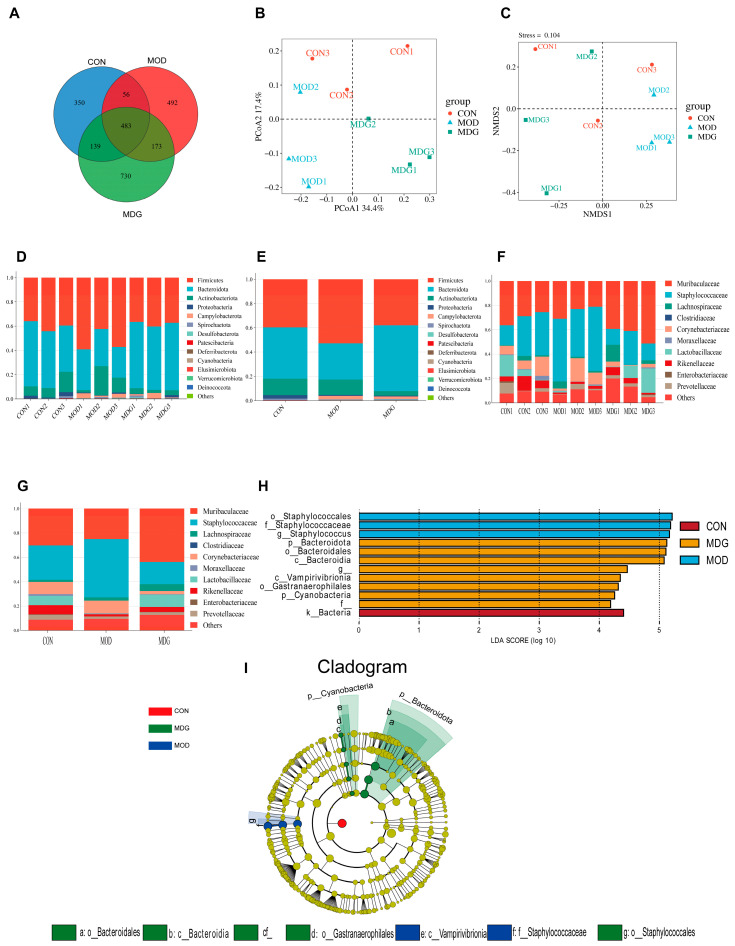
Effect of SA on DON-Induced Gut Microbiota Dysbiosis. (**A**) Venn diagram illustrating shared and unique operational taxonomic units (OTUs) across experimental groups. (**B**) Principal coordinates analysis (PCoA) of microbial communities. (**C**) Non-metric multidimensional scaling (NMDS) analysis of study groups. (**D**) Phylum-level taxonomic composition across groups. (**E**) Phylum-level community distribution in murine microbiota. (**F**) Family-level microbial structure among groups. (**G**) Family-level community distribution in murine microbiota. (**H**,**I**) LEfSe analysis of differentially enriched taxa.

Fecal microbiota profiles across experimental groups were assessed via 16S rRNA sequencing of cecal content. Venn diagrams visualized shared and unique operational taxonomic units (OTUs) among groups (Figure 5A). Principal coordinates analysis (PCoA) (Figure 5B) and non-metric multidimensional scaling (NMDS) (Figure 5C) based on Bray–Curtis distances revealed substantial alterations in overall microbial composition induced by DON exposure compared to the CON. Notably, two-week SA administration significantly shifted microbial profiles in treatment groups.

At the phylum level (Figure 5D), the MOD exhibited a significantly increased relative abundance of *Firmicutes* and decreased abundance of *Bacteroidota* versus CON. These compositional changes were markedly reversed by SA treatment in the MDG. On the family level (Figure 5F,G), DON exposure elevated *Staphylococcaceae* abundance while reducing *Muribaculaceae,* which was similarly ameliorated in the MDG.

LEfSe analysis (LDA score >2.0) further identified differentially enriched taxa (Figure 5H,I). The MOD showed significant enrichment of potentially pathogenic taxa, including *Staphylococcaceae* (LDA = 5.67), *Deferribacterota* (LDA = 4.32), and Bacillus (LDA = 3.41). Conversely, MDG promoted proliferation of beneficial bacteria, particularly *Bacteroidota* (LDA = 5.67), *Muribaculaceae* (LDA = 5.12), and *Prevotellaceae_NK3B31_group* (LDA = 4.54). These microbiota shifts correlated significantly with key intestinal barrier functional markers. Critically, SA pretreatment effectively counteracted DON-induced dysbiosis.

## 4. Discussion

Following ingestion, DON is primarily absorbed via the jejunum [23]. Exposure to DON alters gene expression profiles, particularly those encoding cytokines, while disrupting genes involved in nutrient transport, barrier function, and mitochondrial integrity, ultimately compromising cellular homeostasis [24]. Additionally, DON induces redox imbalance in the gut due to excessive ROS generation and diminished antioxidant capacity [25]. Therefore, enhancing intestinal antioxidant defenses represents a promising strategy to mitigate DON toxicity. In this study, we evaluated SA’s protective effects against DON-induced oxidative stress, aiming to develop novel intestinal protectants.

To assess intestinal damage severity, we measured the jejunal villus height-to-crypt depth ratio (V/C ratio), a well-established morphological indicator of absorptive function [26]. A significant V/C ratio reduction in the MOD indicated compromised structural integrity and nutrient absorption [27]. Elevated DAO and D-LA levels—biomarkers of epithelial damage correlating with gut hyperpermeability—were observed in MOD mice [26,28]. Pro-inflammatory cytokines (TNF-α, IL-6, IL-1β) were significantly upregulated in MOD jejunum, driving barrier disruption through distinct mechanisms [29]: TNF-α induces epithelial apoptosis [30], IL-1β activates inflammatory cascades [31], and IL-6 amplifies immune infiltration [32]. SA intervention suppressed these cytokines, consistent with its anti-inflammatory properties [33].

Intestinal barrier damage is intrinsically linked to tight junction (TJ) dysfunction [34]. Our TEM analysis revealed ultrastructural alterations in DON-exposed jejunum, including disrupted TJ architecture and mitochondrial swelling, indicative of severe oxidative stress-induced barrier damage [35]. SA reversed DON-induced downregulation of key TJ components (ZO-1, Claudin-1, Occludin) at both transcriptional (qPCR) and translational (WB) levels [36]. TJ complexes constitute the gut’s physical barrier, regulating paracellular permeability and preventing pathogen translocation [37]. Crucially, TJ proteins also modulate redox signaling [38], and their downregulation is a recognized biomarker of epithelial damage [39]. Emerging evidence indicates bidirectional TJ-oxidative stress crosstalk: TJ disruption exacerbates ROS generation, while oxidative stress impairs TJ assembly [40]. This interplay underpins SA’s protection via the Nrf2/HO-1 pathway.

Oxidative stress is central to DON-induced enterotoxicity. DON exposure elevates MDA levels while suppressing antioxidant enzymes (SOD, T-AOC, GSH), ultimately causing oxidative damage and barrier impairment [41]. SA, a potent antioxidant, significantly reduced serum MDA and enhanced SOD, T-AOC, and GSH activities in DON-exposed mice. The Keap1/Nrf2 axis is the primary defense against oxidative stress [42]. Under basal conditions, Keap1 binds Nrf2, targeting it for ubiquitin-mediated degradation. During oxidative stress, Nrf2 dissociates from Keap1, translocates to the nucleus, binds antioxidant response elements (AREs), and activates cytoprotective genes (e.g., HO-1, NQO1) [43]. However, under severe oxidative stress, Nrf2 degradation occurs via the β-TrCP-GSK-3β axis independently of Keap1 [44]. Our data show that DON downregulated Nrf2, Keap1, HO-1, and NQO1 expression, whereas SA reactivated the Nrf2/HO-1/NQO1 pathway. Intriguingly, SA did not downregulate Keap1 but maintained it at CON levels. This suggests that SA attenuates DON-induced oxidative stress via Keap1-independent Nrf2 activation—a conclusion supported by emerging evidence [45].

The 16S rRNA sequencing results in this study demonstrate that DON exposure significantly altered the gut microbiota structure in mice, characterized by an increased ratio of *Firmicutes* to *Bacteroidota* at the phylum level and enrichment of potentially pathogenic bacteria such as *Staphylococcaceae* and *Bacillus*. SA treatment effectively reversed these microbial dysbiosis patterns, promoting the abundance of beneficial bacteria, including *Muribaculaceae* and *Prevotellaceae_NK3B31_group*, while restoring beta-diversity profiles toward the control group. These shifts suggest that the protective effects of SA against DON-induced intestinal injury may be partially mediated through gut microbiota modulation.

Previous studies have established that gut microbiota play pivotal roles in maintaining intestinal barrier integrity, regulating bile acid metabolism, and suppressing inflammatory responses [46,47]. For instance, supplementation with *Lactobacillus rhamnosus GG* (LGG) ameliorates microbiota homeostasis and reduces toxin-associated enteropathy [48]. Consistent with these findings, SA intervention in our study not only restored microbial balance but also significantly enhanced jejunal tight junction protein expression and activated the Nrf2/HO-1/NQO1 signaling pathway. This indicates that microbiota remodeling may synergistically confer protection through improved barrier function and antioxidant capacity.

Chemically, SA is a small-molecule organic acid with high bioavailability. While readily absorbed, it may indirectly modulate microbiota composition by altering the intestinal microenvironment. The existing literature suggests that phytochemicals can reshape microbial communities through pH modulation, provision of metabolic substrates, or pathogen inhibition [49]. Furthermore, microbiota-derived metabolites such as short-chain fatty acids (SCFAs) have been shown to regulate oxidative stress and inflammation via the gut-liver-immune axis [50,51,52]. Although SCFA levels were not directly measured in this study, the SA-induced proliferation of beneficial bacteria implies potential involvement of these metabolites in mitigating DON toxicity—a hypothesis warranting further experimental validation.

Collectively, these findings support the hypothesis that SA confers protection against DON-induced jejunal barrier damage, potentially through activating the Nrf2/HO-1/NQO1 pathway and modulating gut microbiota. Notably, we observed an inverted U-shaped dose–response relationship across LDG, MDG, and HDG groups, characteristic of non-monotonic pharmacological effects. However, the precise mechanisms underlying this biphasic response remain elusive, representing a current limitation of our study. It is also essential to identify the specific active components responsible for the observed effects throughout this research. Furthermore, while SA-mediated activation of the Nrf2/HO-1/NQO1 axis was demonstrated herein, future investigations are warranted to determine whether this occurs through the GSK-3β pathway. Regarding microbial analyses, the dependence of SA’s protective effects on gut microbiota remodeling has not been functionally validated through approaches such as fecal microbiota transplantation (FMT). Additionally, given that gut microbiota homeostasis is influenced by multiple confounding factors (e.g., diet, environmental conditions, and host physiological status), subsequent studies should integrate multi-omics methodologies to elucidate the precise molecular mechanisms governing SA-microbiota-host interactions.

## 5. Conclusions

DON causes jejunal injury in mice, while SA mitigates this damage to the intestinal barrier and modulates gut microbiota, potentially via activating the Nrf-2/HO-1/NQO1 pathway.

## Figures and Tables

**Table 1:** All specific upstream and downstream primer sequences for qRT-PCR.

Gene Target	Primer (5′–3′)	Size	Sequence Number
β-actin	F: TATAAAACCCGGCGGCGCA R: GTCATCCATGGCGAACTGGTG	118	NM_007393.5
Keap1	F: GCCCCGGGACTCTTATTGTG R: TTAGGGGCCCCGCCAT	101	NM_001110305.1
Nrf2	F: ACTACAGTCCCAGCAGAGTGAT R: TCACACACTTTCTGCGTGCT	109	NM_001399226.1
NQO1	F: TCTCTGGCCGATTCAGAGTG R: CCAGACGGTTTCCAGACGTT	145	NM_008706.5
HO-1	F: ACCTTCCCGAACATCGAGACAG R: CAGCTCCTCAAACAGCTCAATG	150	NM_010442.2
Claudin 1	F: TGGGGCTGATCGCAATCTTT R: CACTAATGTCGCCAGACCTGA	137	NM_016674.4
Occludin	F: CCCTCTTTCCTTAGGCGACA R: CCCAAGATAAGCGAACCTGC	95	NM_001360536.1
ZO-1	F: CTTCCCGGACTTTTTGTCCCA R: CATTGCTGTGCTCTTAGCGG	220	NM_001163574.2
TNF-α	F: ACCCTCACACTCACAAACCA R: ATAGCAAATCGGCTGACGGT	212	NM_001278601.1
IL-1β	F: TGCCACCTTTTGACAGTGATG R: TGATGTGCTGCTGCGAGATT	138	NM_008361.4
IL-6	F: AGACAAAGCCAGAGTCCTTCAG R: TGTGACTCCAGCTTATCTCTTGG	77	NM_001314054.1

## Data Availability

Data are contained within the article.

## Abbreviations

The following abbreviations are used in this manuscript:

SA	Shikimic Acid
DON	Deoxynivalenol
